# Non-Lethal Blood Sampling of Fish in the lab and Field With Methods for Dried Blood Plasma Spot Omic Analyses

**DOI:** 10.3389/fgene.2022.795348

**Published:** 2022-03-24

**Authors:** S Pollard, JC Anderson, F Bah, M Mateus, M Sidhu, DBD Simmons

**Affiliations:** Aquatic Omics Laboratory, Department of Biology, Ontario Tech University, Oshawa, ON, Canada

**Keywords:** non-lethal, blood sampling, fish, plasma, proteomics, metabolomics, dried blood spots (DBS), dried plasma spots (DPS)

## Abstract

There is global acknowledgment that humane methods in animal research are a priority, but few environmental effects monitoring programs use nonlethal methods for fish. The goal of the present study was to determine the impacts of sampling small volumes of blood in larger-bodied fish on survival and healing. In addition to evaluating survival following blood sampling, we evaluated the utility of dried blood spots as an alternative for sample processing and storage in the field. In our approach, we housed 80 rainbow trout (*Oncorhynchus mykiss*) in our flow-through aquatic facility. We then anaesthetized using MS-222 and sampled 1 μl/g bw of blood via puncture of the caudal vasculature. We tested four different post-blood sampling treatments on the puncture wound: 1. application of liquid bandage; 2. a swab of betadine; 3. a swab of fish mucous; and 4. compared survival outcomes to a group where no post-treatment was performed (negative control). Overall, we observed 90% survival among all treatments, with the most effective approach being the negative control (100% survival). Based upon these results, we repeated the blood sampling with no-post treatment by housing 20 rainbow trout (not previously tested upon) in cages at a nearby creek and monitored survival for 2 weeks post sampling. The survival rate was 95% with full healing of the puncture site in all subjects. In addition to this, we tested the efficacy of dry blood spotting on proteomic, lipidomic and amino acid analysis as an alternative method for blood sample processing and storage. It was found that dried plasma spotting using parafilm in conjunction with a modified Bligh-Dyer extraction offered the best balance for good recovery of protein, lipid and amino acids relative to wet plasma and Noviplex dried plasma spot cards. In this article, we will present the detailed results of these combined studies and describe what we have determined to be the safest non-lethal blood sampling protocol.

## 1 Introduction

Blood is composed of a complex mixture of different cell types, ions, gases and a multitude of biomolecules including RNAs, proteins and metabolites (Duman et al., 2019; Lawrence et al., 2020; Seibel et al., 2021). It is from this diverse medium, that hundreds of millions of different variables can be measured for biomonitoring purposes (Seibel et al., 2021). Additionally, blood circulates through the entire body of an organism, collecting information from different biological systems, providing a holistic perspective on organismal health. Sampling blood from fish has long been used in aquaculture and environmental monitoring practices as a non-lethal and useful indicator of physiology, disease and chemical contamination (Teffer and Miller, 2019; Andrew et al., 2021; Seibel et al., 2021). However, there is concern as to whether sampling blood from fish, especially threatened wild fish species, can cause physical harm or lead to mortality. There is a dearth of published literature with empirical results that demonstrate survival of fish post blood sampling, which is important information required for ethical decision making and experimental design.

Several methods of blood sampling exist for fish including tail ablation, heart puncture, dorsal aorta puncture and puncture of the caudal vasculature (Duman et al., 2019). Sampling blood via the caudal vasculature is well described by Lawrence et al. (2020) and generally considered to be the safest, as it minimizes handling times and can be administered across the greatest range in size of fish. It is advisable to use anesthesia when sampling blood from fish, in order to minimize movement during sampling which could cause serious injury to the animal (Barton and Iwama, 1991). Traditional methods of anesthesia include chemical anesthetics such as tricaine methanesulfonate and clove oil, however these methods do not allow for quick induction or recovery times of the fish, and are typically subject to impractical depuration periods from regulatory bodies (i.e., the U.S. Food and Drug Administration and Health Canada) prior to release back into the field (Health Canada, 2010; Trushenski et al., 2013). Electro-anesthesia/electro-sedation is a technique that has been widely used for decades in aquaculture and in fisheries for temporary immobilization and handling (Robb and Roth, 2003; Reid et al., 2019). It is ideal for field handling of fish due to quick induction and recovery times, without the need for depuration periods as is needed with chemical anesthetics (Keep et al., 2015; Abrams et al., 2018; Reid et al., 2019).

Another important consideration is the volume of blood which can be taken from fish without causing significant impacts to health from hemodilution (Duff and Olson, 1989; Fazio et al., 2015; Lawrence et al., 2020). The Canadian Department of Fisheries and Oceans recommends sampling blood for non-lethal practice should remove no more than 0.1% of body mass in fish greater than 200 g (Canadian Department of Fisheries and Oceans, 2004). Of course, depending on the type of analysis, the volume of blood required can vary greatly and it is advisable to take this into consideration for the size of fish being used in a given experiment. A general rule is that blood volume in Teleostei is approximately 3–7% of body mass depending on the species involved (Smith, 1966; Tort et al., 1991). In addition to this, many hematological analyses involve separation of plasma from the blood, losing 30–50% volume which approximately doubles the volume of blood required (Lawrence et al., 2020). Thus, it is a challenge when sampling blood from fish that are less than 100 g in body mass as the amount of plasma required for some analyses can exceed the 0.1% volume per bodyweight recommendation.

Dried blood spots (DBSs) and dried plasma spots (DPSs) are a promising, alternative means of processing blood samples obtained in the field as it forgoes the requirements for centrifugation and immediate cold storage, while only requiring low volumes of blood (i.e., 15–25 µl of whole blood for proteomics). Several studies have found plasma separation cards to be effective at achieving good analyte stability and recovery for analyses involving RNAs, proteins and metabolites (Aristizabal Henao et al., 2016; Björkesten et al., 2017; Carmona et al., 2019). Thus, these alternatives to post sampling treatment may prove to be an effective alternative for non-lethal health monitoring of fish in the field especially when cold storage and centrifugation are not immediate options.

Many studies have sampled blood via cannulation of the dorsal aorta or via caudal puncture in different fish species, and have observed survival after days to months (Lowe-Jinde and Niimi, 1983; Foo and Lam, 1993; Mazik et al., 1994; Lo et al., 2003; Djordjevic et al., 2012). Cook et al., 2011 even observed survival in largemouth bass released after a year in a private research lake. However, there is no published study that we are aware of, which has explicitly measured and reported survival outcomes following blood sampling. Thus, the primary purpose of the current study is to evaluate the impact of blood sampling via caudal puncture on the healing and survival of fish, and to evaluate different methods for collecting and storing plasma samples from blood. The study was conducted in three parts; 1) blood was sampled from rainbow trout in the laboratory and several post-sampling treatments were administered to test any improvements on healing and survival; 2) A follow-up experiment was conducted, evaluating the effect of blood sampling on the survival of caged rainbow trout in the field; and finally 3) we evaluate the utility of dried blood spots for metabolomic and proteomic analysis.

## 2 Materials and Methods

### 2.1 Blood Sampling in the Laboratory

**FIGURE 1:**
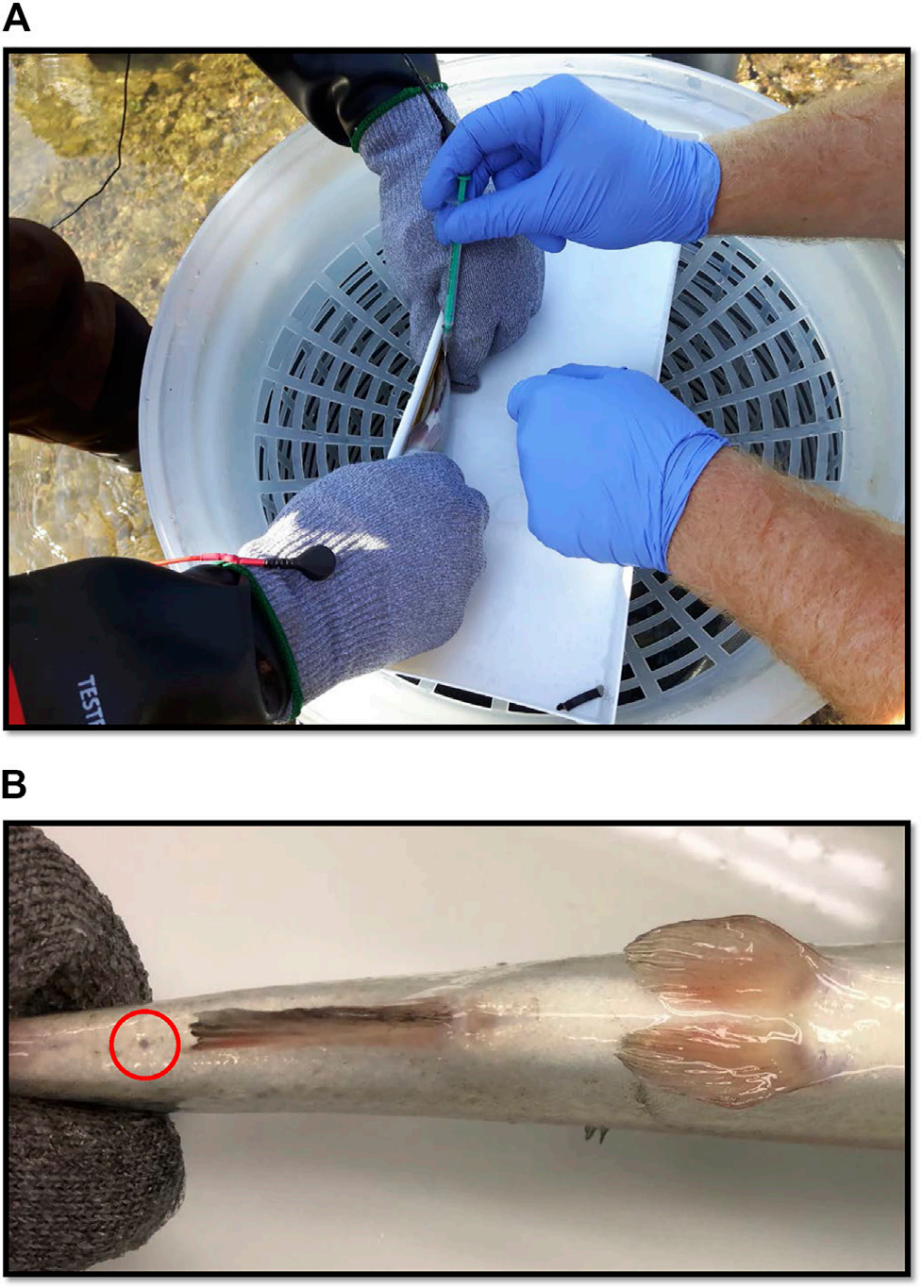
**(A)** Blood being sampled from a rainbow trout (100 g) in the field with a needle and syringe. **(B)** Small scar (circled in red) which occurred in 7 of 20 blood sampled fish in the field. The mark is the only discernible feature between healthy controls and fish whose blood had been sampled. Photograph was taken after 3-weeks of recovery post-sampling.

The animal care committee at Ontario Tech University reviewed and approved the use of animals in the proceeding experiment under animal use protocol 15031. We housed 80 rainbow trout (approximately 200–300 g) in four 1000-L flow-through tanks (20 fish per tank) with five turnovers per day, using de-chlorinated municipal water at a temperature of 12°C, and a photoperiod of 16:8 light:dark. We kept 5 fish from each treatment in each of the four tanks (20 fish per tank), making each tank a technical replicate with five pseudo replicates. In order to keep track of each treatment, we tagged fish in the dorsal muscle tissue with a floy tag; each having unique IDs. The post-sampling treatments involved application of a liquid bandage, a Betadine^®^ swab, a swab containing the fish’s own mucous, or no post-treatment (pressure-only). We allowed the fish to acclimate in the lab for several weeks prior to blood sampling and fed them a diet of 1.6% bw (Corey Aquafeeds, Optimum™ 3.0 mm size pellets) every other day throughout the study. We anesthetized fish using tricaine methanesulfonate and measured body weight, length, and positioned the fish in a wet, foam V-trough to sample blood from the caudal vasculature, with needle entry posterior to the anal fin (Figure 1A). We withdrew approximately 1 µl of blood per gram of body weight using a 22-gauge needle and a heparinized syringe. We applied pressure and waited a minimum of 30 s to stop bleeding prior to administering each post-blood sampling treatment. We let each fish recover from the anesthetic in an aerated bucket and then transferred them to their original holding tanks. Previously, we have found the heparinization of the needle is not necessary and can cause excessive bleeding even after applying pressure to the wound for 30 s.

One week after sampling blood, we lightly anesthetized the fish and examined them. We photographed each fish and manually recorded notes on condition and healing. On week 2, we re-examined the fish (day 14), but this time we recorded notes for each fish, and only took photographs of fish that were not fully healed. At this point we pooled together the healthy (apparently healed fish) into two tanks, and kept a third tank for the fish that needed to be re-examined (ones that had not yet fully healed, or showed signs of necrosis or infection). We monitored the fish for two more weeks–putting healthy ones back into the “healthy population” tanks and recorded the progress and/or mortality of the remaining fish.

### 2.2 Blood Sampling in the Field

The animal care committee at Ontario Tech University reviewed and approved the use of animals in the proceeding experiment under animal use protocol 15401. We housed 40, 150-g rainbow trout and allowed them to acclimate in the lab for 2-weeks and subjected them to the same housing conditions as described in Section 2.1. Following acclimation, we transported fish to Oshawa Creek on 12 May 2021 into two 45 cm × 45 cm × 122 cm, rectangular cuboid cages constructed out of 1.9 cm diameter PVC pipe, 3-way elbow joints and black vinyl fencing material with 2.5 cm diameter holes, fastened with zip-ties. We determined the water temperature of the creek at the time of transport to be 11°C; one degree colder than the temperature in the laboratory. We observed one mortality the morning after transport and immediately replaced it. Positioning of the cages was side by side in a natural hole formed within a bend of the creek which had good stream flow. We monitored water temperature in each of the cages using a HOBO™ UA-002-64 pendant which recorded creek temperature every 20 min throughout the study (Supplementary Figure S1). We monitored fish health daily in addition to taking manual measurements of temperature and pH. Following a 13-days acclimation period in the field, we captured fish via hand held net and sampled blood using the same technique as described in the pilot study. We used the “pressure-only” post-treatment which was compared to a control group wherein no blood would be sampled. We used electric fish handling gloves (Smith-Root) to sedate fish instead of tricaine methanesulfonate because it allows for quick handling and recovery times in the field. We allowed the fish to recover for 2 min in a recovery bath prior to being returned to their original cage. In order to eliminate the effects of handling on mortality, we handled control fish in the same manner as the blood sampled fish, including the use of electrofishing gloves. In order to eliminate the possibility of caging effects, we sampled blood from ten fish from each cage. We decided that tagging was not necessary because the wound from the blood sampling would be observable for 1–2 weeks in the case of a mortality. We observed one mortality in a fish the day after sampling and did not replace it (images available in the Supplementary Material). Upon examination, there were no outward signs of trauma or infection suggesting that the mortality could have been due to handling related stress alone.

### 2.3 Statistical Analysis of Survival Data

We compiled all survival data collected from the laboratory and field sampling studies into 2 × 2 contingency tables. With regards to the laboratory pilot study, the “pressure-only” treatment served as the negative control which was compared to the other post-sampling treatments.

We statistically analyzed data using the R software environment (V 4.0.5) with the “Exact” package (V. 2.1) installed. We conducted a Barnard’s CSM test on survival data because it is determined to be a more appropriate test with greater statistical power than the Fisher’s exact test for experiments of one margin fixed design (Lydersen et al., 2009). More specifically, we used the “exact.test” function with the arguments: alternative = “greater”, method = “CSM”, conf.in = TRUE) which specified the use of a one-tailed Barnard’s test because the frequency of mortality was only expected to increase from a frequency of zero. The program also calculated a 95% confidence interval for each experimental group using the argument conf.in = TRUE. With respect to the laboratory pilot study, we corrected the p-values obtained for each post-sampling treatment for multiple comparisons using the p.adjust function available in base R which performs a Benjamini-Hochberg FDR correction. The outputs of these statistical tests are available in the Supplementary Materials.

### 2.4 Sample Preparation

**FIGURE 2:**
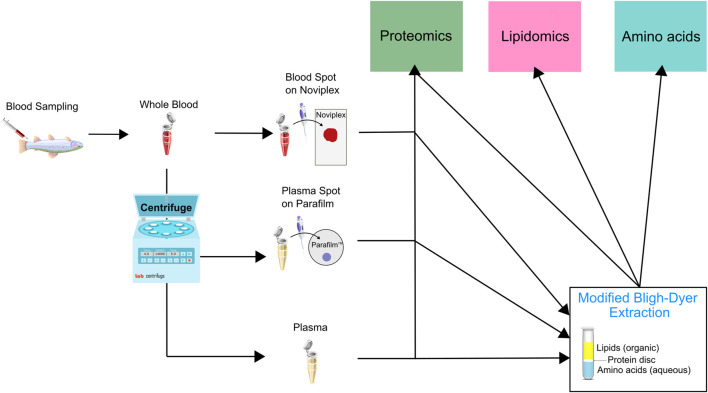
Schematic illustrating the sample preparation pipeline used for untargeted proteomic, lipidomic and analysis using LC MS/MS. Briefly, we pooled whole blood from 20 rainbow trout and underwent sample preparation through three initial channels which were either a blood spot on Noviplex (NP), plasma spot on parafilm (PF) or just regular wet plasma (WP). The three types of samples were then run through two channels which were 1) directly entering the proteomics sample extraction or 2) entering a modified Bligh-Dyer extraction which was then run through the three different extraction protocols (proteomics, lipidomics and amino acids). The PF Disc and WP Disc are used to refer to proteomics results from samples prepared using the modified Bligh-Dyer extraction.

In order to minimize biological variation, we pooled whole blood samples obtained from 20 different fish. We then applied 60 μl of the blood onto Noviplex™ Duo Plasma Prep Cards (NP) and the blood stayed on the membrane for 3 min before the top layer was peeled off and allowed to air-dry for 15 min. We stored the plasma prep cards in their original packaging at room temperature until further processing. A schematic which outlines the different sample preparation workflows is available (Figure 2).

We centrifuged the remaining blood at 2000 × g for 10 min at 4°C to separate the plasma. We then spotted ten, 15 μl parafilm (PF) spots with the plasma and dried them under a fume hood. Once dried, we stored PF DPSs at room temperature in Ziploc^®^ bags until further processing. We transferred the remaining plasma to cryogenic vials, flash froze them in liquid nitrogen and stored them at −80°C until further analysis.

#### 2.4.1 Recovery of Plasma Proteins From Dried Spots

We transferred the NP collection disks into plastic, low retention microcentrifuge tubes along with 25 µl of ammonium bicarbonate (AB) buffer. We agitated the tubes using a VWR^®^ Mini Shaker at a speed of 165 rpm for 10 min, repeated the process 2 more times and then transferred the supernatant from the three agitations to a second low retention microcentrifuge tube. In total, we used 40 discs from a pooled blood sample, making 10 technical replicates of 75 µl each containing approximately 15 μl of plasma (based upon the manufacturer’s description). With respect to the PF DPSs, we recovered plasma proteins by adding 25 µl of AB buffer with agitation to resuspend dried proteins. In this case, we repeated the process only twice, ending with a total volume of 50 µl of AB buffer. We then dried down all samples using a centrifugal evaporator (Savant™ SpeedVac™ SPD1030) so that they all had approximately 15 μl of volume.

#### 2.4.2 Modified Bligh-Dyer Metabolite and Protein Extraction

We quenched all of the samples with 15 μl of cold HPLC-grade methanol. We then used a modified Bligh-Dyer extraction method. Briefly, we sonicated samples for one-second and repeated 5 times; and then added 15 μl chloroform, 30 μl Milli-Q™ water, and 15 μl chloroform, vortexing for 20 s after each addition. We then centrifuged samples at 2,400 × g for 30 min at 4°C, which resulted in three visually distinct layers–an upper aqueous layer containing the amino acids, a lower organic layer containing the lipids, and a middle layer containing the protein disk. We then separated each liquid layer by transferring 15 μl of sample to a new tube. The protein disks were air dried and re-suspended in 50 µl of AB buffer. The specific methods for each set of analytes from each plasma fraction is described further below.

#### 2.4.3 Protein Preparation

We prepared samples for non-targeted proteomic analysis as described previously by Simmons et al. (2012) and will be summarized in brief herein. We reduced protein disulphide bonds by adding 2.65 µl of 100 mM of Tris(2-carboxyethyl) phosphine (TCEP) to 15 μl of wet plasma resuspended in 35 μl of AB buffer, or the resuspended protein disks that were in 50 µl of AB buffer. We then alkylated proteins by adding 2.8 µl of 200 mM of iodoacetamide and digested by adding 50 µl of 20% formic acid and incubating for 30 min at 115°C. We then evaporated the digested samples to near dryness using the centrifugal evaporator and then reconstituted in 20 µl of 95% Milli-Q™ water, 5% acetonitrile, 0.1% formic acid. In order to remove debris, we centrifuged samples again for 10 min at 14,000 × g and transferred the supernatant into chromatography vials containing 250 µl polypropylene conical inserts for subsequent analysis by high performance liquid chromatography tandem mass spectrometry (HPLC-MS/MS).

The Grand Average of Hydropathy (GRAVY) value was calculated for all proteins identified in the dataset using the “Sequence Manipulation Suite” (Stothard, 2000). The calculated GRAVY scores for each rainbow trout protein is included as a file in supplementary.

#### 2.4.4 Lipids

We spiked the organic layer obtained from the modified Bligh-Dyer extraction (15 µl) with 7.5 µl of SPLASH^®^ LIPIDOMIX^®^ Mass Spec Standard, and then evaporated the samples until near dryness using centrifugal evaporation. We then re-suspended the samples in 10 μl of 5:1:4 of isopropanol: methanol:5 mM ammonium acetate solution and vortexed for 2 min. We also prepared a composite sample by pooling 2 μl of each sample for quality control and for iterative tandem mass spectrometry to collect comprehensive product-ion spectra for subsequent use in lipid annotator software which conducts feature identifications. All samples were transferred into chromatography vials containing 250 µl polypropylene conical inserts for subsequent analysis by HPLC-MS/MS.

We performed instrumental analysis using an Agilent 6545 Series quadrupole time-of-flight (Q-TOF) coupled to an Agilent Infinity 1260 HPLC. More specifically, samples were separated using a high pH resistant C18 column (Agilent InfinityLab Poroshell HPH-C18, 2.1 × 100 mm, 2.7 μm) for detection in negative mode, with 5:1:4 isopropyl alcohol/methanol/water with 5 mM ammonium acetate and 0.1% formic acid as mobile phase A and 99:1 isopropyl alcohol/water with 5 mM ammonium acetate and 0.1% formic acid as mobile phase B (detailed instrumental methods are available in Supplementary Information).

#### 2.4.5 Amino Acids

We spiked the 15 μl aqueous fraction obtained from the modified Bligh-Dyer extraction with 7.5 µl of a mixed stable isotope labelled standard (Canonical Amino Acid Mix, Cambridge Isotope Laboratories) and then evaporated the samples in a centrifugal evaporator until near dryness. The samples were resuspended in 10 μl of 90% HPLC-grade Acetonitrile and 10% Milli-Q™ water. A pooled composite was also prepared by adding 2 μl of each sample to a separate vial for quality control and iterative tandem mass spectrometry to collect comprehensive product ion spectra for each amino acid. All samples were transferred into chromatography vials containing 250 µl polypropylene conical inserts for subsequent analysis by HPLC-MS/MS.

Analytes were separated on a HILIC zwitterion column (Agilent InfinityLab Poroshell 120 HILIC-Z, 2.1 × 150 mm, 2.7 μm, PEEK lined) using an Agilent Infinity 1260 HPLC coupled to an Agilent 6545 Q-TOF for detection with 10 mM Ammonium formate with 0.1% v/v formic acid as mobile phase A, and 90% acetonitrile in 10 mM Ammonium formate with 0.1% v/v formic acid as mobile phase B (see detailed instrumental methods are available in Supplementary Material).

#### 2.4.6 Analysis of Spectral Data

For proteins, we used Agilent Spectrum Mill Software (Version 6.0) to sequence peptides and search those against the Rainbow Trout reference proteome (Uniprot, proteome ID#: UP000193380, downloaded January 2020). Proteins were manually validated and included in the dataset if they had at least one peptide with a score > 6, and percent spectral intensity (% SPI) greater than 70.

We subsequently exported spectral files (.d, pep.xml, .MZml and .aph) from Spectrum Mill and imported them into Skyline targeted proteomics software (V20.2) for further processing (Schilling et al., 2012). We used the data dependent acquisition, MS1 filtering import wizard to create a spectral library of non-redundant MS/MS identifications for each protein identified across the batch. All settings were kept at default except the ones mentioned herein. Firstly, the rainbow trout reference proteome was imported in FASTA format for *in silico* digestion. The number of precursor charges were set from 2-6, maximum missed cleavages were set to 5 and a formic acid (D| -) C-terminal and (- | D) n-terminal was used for digestion.

Once the spectral library was created, minimum and maximum peptide lengths were set to 5–30 amino acids with no exclusion of N-terminal amino acids under the “filter” tab within “peptide settings”. The precursor mass analyzer was set to TOF and DDA as the acquisition method under the “full-scan” tab within “transition settings”. All the results (.d files) were imported into skyline and additional protein identifications were made using the library. We exported our results using the MPP APR report tool which formats all the results in a tabular, text format.

We used Lipid annotator software (V 1.0, Agilent) to create a personal compound database and library (PCDL; PCDL Manager B08.00) out of non-targeted MS/MS spectra collected from a pooled composite of the standard-spiked samples for identification purposes using mass and retention time information. We identified and extracted chromatographic peaks from MS-only data using a targeted batch workflow with Agilent Profinder 10.0 software and the custom PCDL database created by Lipid Annotator.

For amino acids, we constructed a PCDL database (PCDL Manager B08.00) using Masshunter, qualitative analysis software from MS/MS spectra obtained from pure, non-labelled and labelled standards that included masses and retention times. Chromatographic peaks were identified and extracted from MS-only data using a targeted batch workflow with Agilent Profinder 10.0 software and the PCDL database.

Statistical analysis of protein and metabolite data was performed using Metaboanalyst 5.0 (Pang et al., 2021). All protein and metabolite data were median normalized, cube root transformed and pareto scaled (*n* = 7). Differences in means were analyzed using a one-way analysis of variance (ANOVA; α = 0.05) with a Tukey’s post-hoc; α = 0.05) and correction of raw p-values for false discovery rate (FDR) using the Benjamini-Hochberg method (FDR < 0.05).

All figures except the venn diagram were constructed using GraphPad Prism 9 software. The Venn diagram was constructed in R using the ggvenn package.

## 3 Results

### 3.1 Blood Sampling in the Laboratory

At the time of tagging, we found no significant differences in length, weight, or condition factor among any of the sub-groups by post-sampling treatment (Supplementary Figures S2–S4). There were also no significant differences in the survival or healing rate among tanks, thus potential tank-effects were ruled-out. Herein, we have pooled together post-treatment subgroups from each tank for statistical analyses.

#### 3.1.1 Betadine Swab Post-Treatment

**FIGURE 3:**
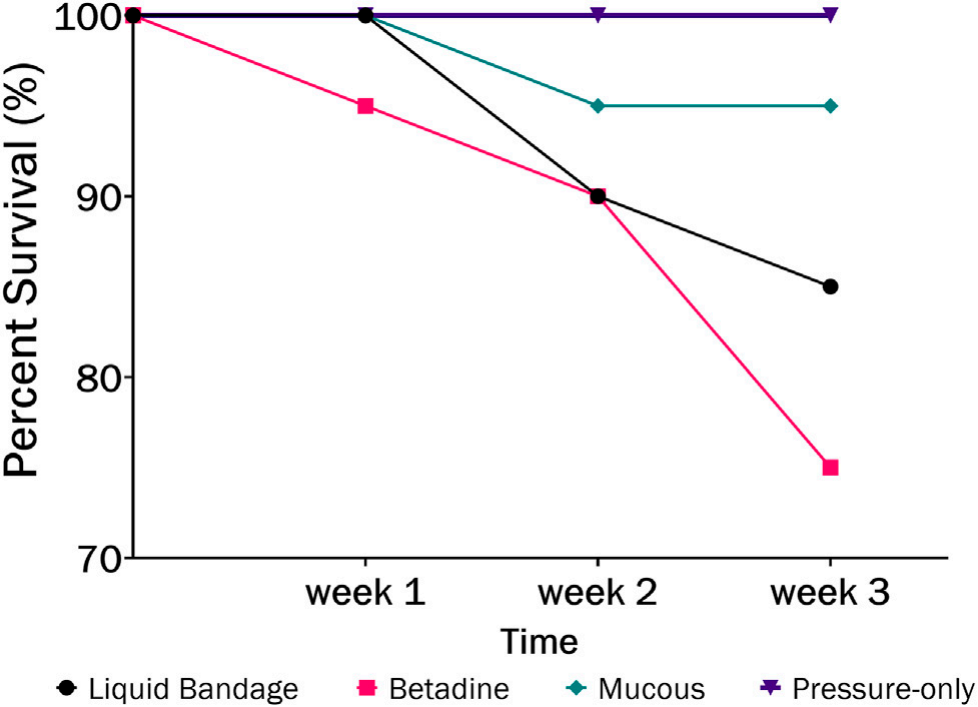
The percent survival of fish after 3-weeks recovery following blood sampling in the laboratory wherein different post-sampling treatments were applied to the wound (liquid bandage: black, Betadine™: red, fish mucous: green, pressure-only: purple).

**FIGURE 4:**
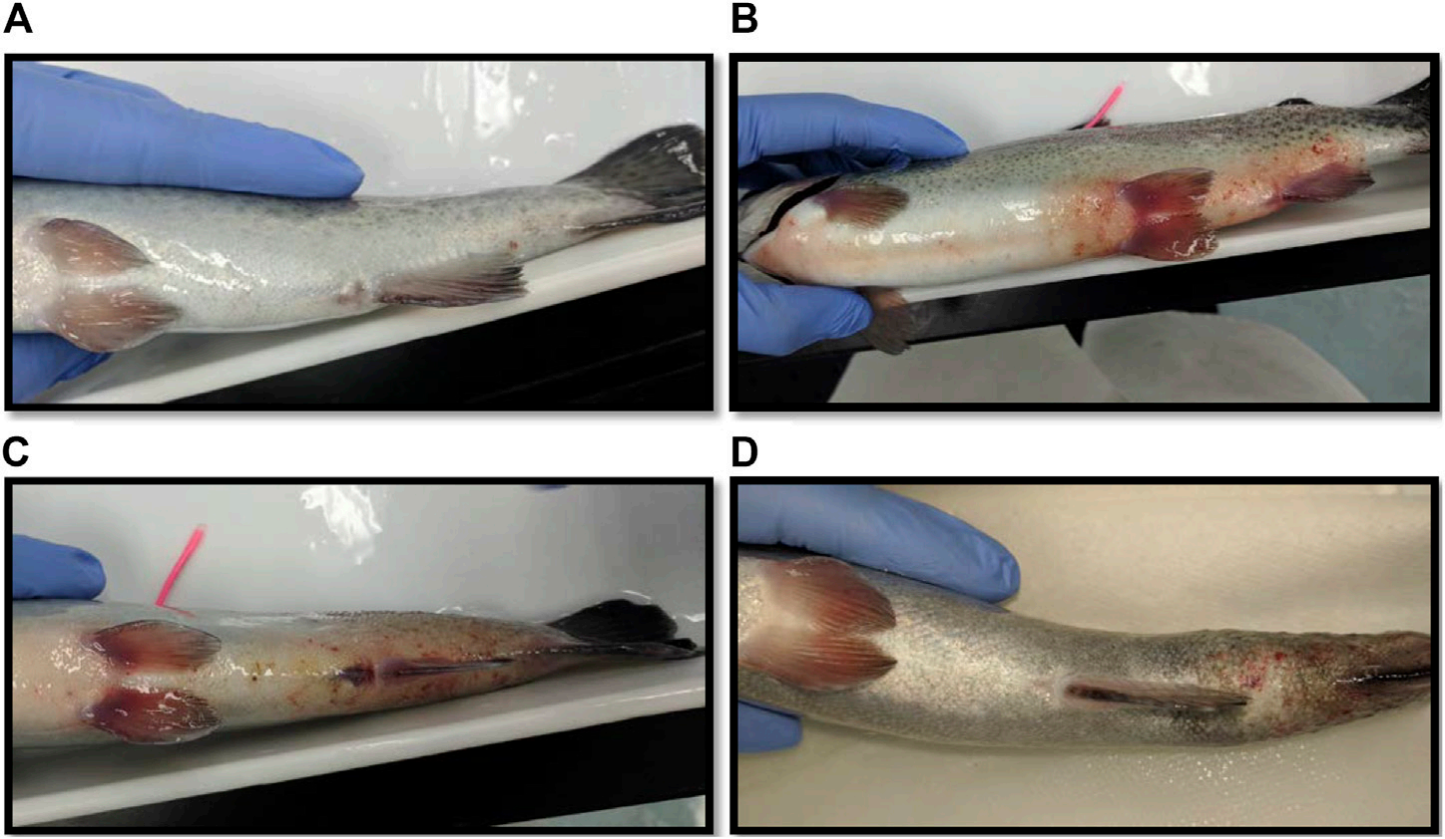
**(A)** Photograph of a fish considered fully healed after sampling blood in the laboratory. **(B)** A fish with a red and swollen abdomen. **(C)** A fish with a red and swollen tail. **(D)** A fish whose tail has become necrotic after blood sampling.

The largest number of fish deaths occurred in the betadine™ swab post-treatment, with the proportion of mortalities being significantly greater than 0 for this post-sampling method (Figure 3; Barnard’s original CSM test, one-tailed, FDR < 0.05). Also, the betadine™ post-treatment had the lowest number of fish recoveries 1 week after sampling, and throughout the duration of the four-week recovery period. One week after blood sampling, 15 fish had healed based upon external observation, 4 fish had swollen tails or abdomens and 1 fish had died (Supplementary Table S1). Generally, unrecovered fish following sampling events exhibited 2 different major symptoms being swollen/red tails and/or swollen/red abdomens which are photographed in (Figures 4A-D; Supplementary Table S1). By the fourth week, there were no more deaths and all remaining fish had healed. Of the five total deaths in this treatment group, 3 were identified after the first week as not having yet recovered, while 2 of the deaths occurred in fish which had no initial signs of trauma or distress.

#### 3.1.2 Liquid Bandage

One week after blood sampling, 17 fish had healed externally, 2 fish had red and/or swollen tails, and 1 fish jumped off the bench during examination and injured its head. By the fourth week, there were no more deaths and the remaining 18 fish had healed. Of the 2 total deaths in this treatment group, 1 was identified after the first week as not having yet recovered, while the other death was most likely related to a head injury and not the blood sampling (although we did not conduct a necropsy to determine exact cause of death.)

#### 3.1.3 Mucous Swab

One week after blood sampling, 15 fish had healed based upon external observation, 5 fish had not healed. In some cases the non-healed fish exhibited abdominal swelling and/or redness closer to the pelvic fins suggesting these symptoms may not be associated with blood sampling (see Figures 4E,F). After 3 weeks, there was one mortality (identified in the first 2 weeks as having a swollen abdomen), and after the fourth week all fish had healed sufficiently and there were no more mortalities. There was no significant difference found between the proportion of fish mortalities in the mucous swab cohort when compared to controls.

#### 3.1.4 Pressure-Only Post-Treatment

This treatment group was the most successful–with no mortalities and the best overall healing success. One week after blood sampling, 18 fish had healed based upon external observation, and 2 fish had swollen abdomens–one of which also had a swollen tail. Two weeks after blood sampling, the same 2 fish were nearly healed, with only minor swelling and redness compared to the previous week. After 3 weeks, all fish had healed sufficiently. There was no significant difference found between the proportion of fish mortalities in the pressure-only cohort when compared to controls.

### 3.2 Blood Sampling in the Field

We observed one mortality in the field approximately 24 h following the blood sampling event, in a fish whose blood had been sampled. There were no external signs of trauma or infection suggesting the mortality is most likely due to general stress associated with handling.

All of the fish were observed to have completely healed 2-weeks after the blood sampling event (Supplementary Figure S5). There were some minor scrapes and sores on 3 fish which were separate from the actual site of puncture and were thus not associated with sampling but with general injuries that can occur from fish handling and transport.

All of the fish were clearly in good health, and the majority of blood sampled fish were indiscernible from controls (Images available Supplementary Materials). The fish were not tagged and so the fact that there were no signs of blood sampling in 11 out of 20 fish suggests the sampling was well tolerated. With respect to the other 9 fish, the sites were fully healed but a small scar was visible at the site of sampling (Figure 1B).

### 3.3 Proteomics

**FIGURE 5:**
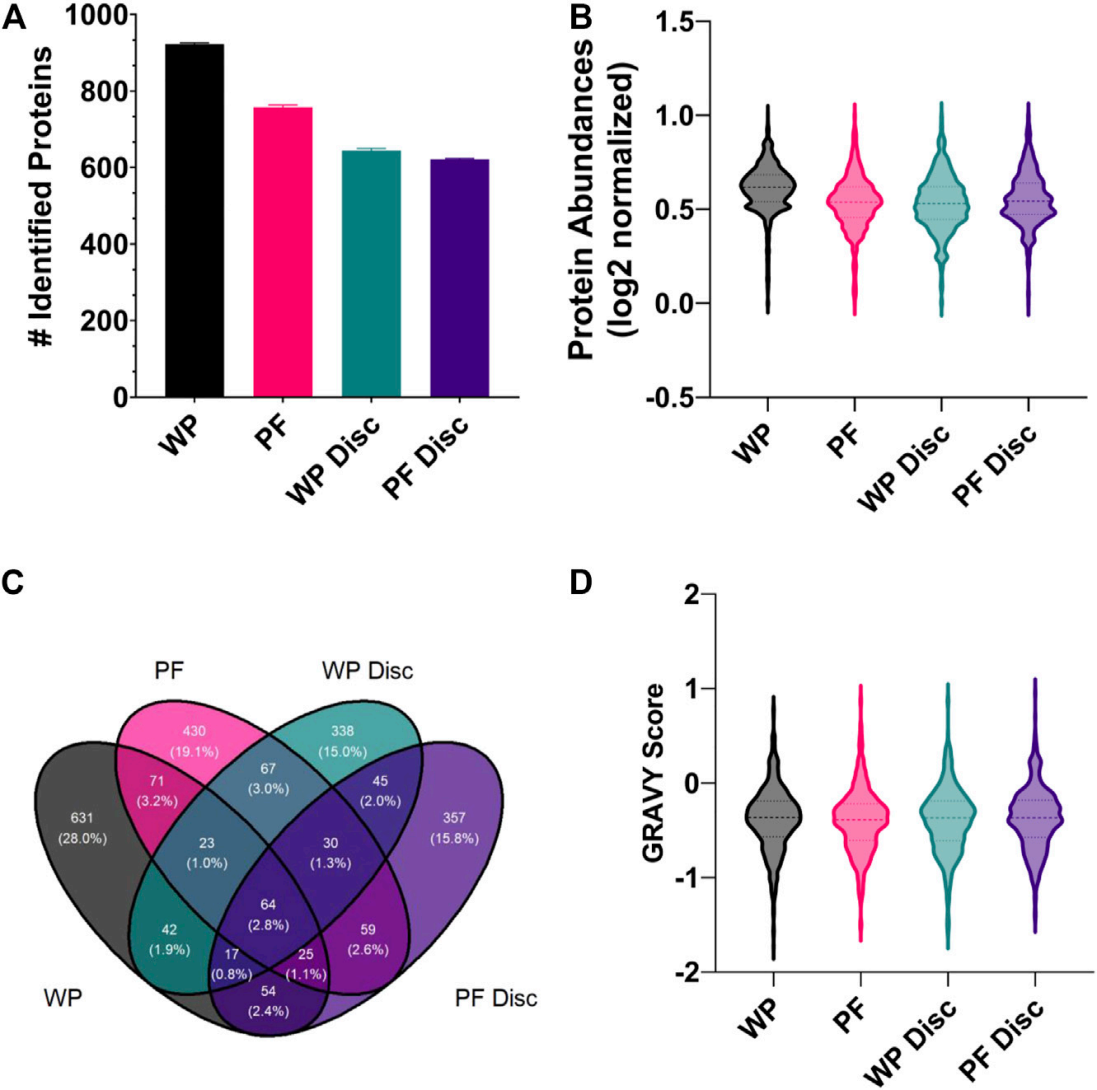
**(A)** The mean number of rainbow trout proteins identified in each sample processing method (SPM). The error bars display standard deviation. Abbreviations: WP (wet plasma); PF (Parafilm). **(B)** A violin plot illustrating the frequency distribution of average protein abundances within each SPM. All protein abundance data was plotted as mean normalized, log2 transformed. Central dotted line within the violin represent the median and outer lines represent quartiles. **(C)** A Venn diagram comparing the total number of identified proteins for each sample processing method (WP, PF, WP Disc, and PF Disc). **(D)** Violin plot illustrating the frequency distribution of GRAVY scores determined for proteins identified within each SPM. The WP Disc and the PF Disc represent the protein discs obtained from the modified Bligh-Dyer extraction. Abbreviation: WP, wet plasma; PF, parafilm; WP Disc, plasma protein disk); PF Disc, parafilm protein disk).

There were no identifiable proteins in the NP samples so we excluded the NP sampling method from statistical analysis. Unsurprisingly, the wet plasma sample processing method (SPM) exhibited the greatest number of protein identifications and average protein recoveries compared to the others (Figures 5A,B). With respect to the average number of protein identifications made, 923, 757, 644, and 621 were made for the WP, PF, WP Disc and PF Disc SPMs respectively. Overall, repeatability of protein identifications across replicates was good being approximately 99% for all SPMs which is likely due to the use of MS1 filtering of mass spectral data in Skyline. Essentially all protein MS/MS identifications are compiled into a library and identifications are made using precursor ion mass and retention time alignment across the entire batch of replicates. The outcome greatly increases repeatability of protein identifications across replicates.

A small proportion of proteins were found to be in common across the different SPMs (Figure 5C). In general, the most populated cross-sections were those unique to each SPM. In order to find potential reasons for differences in types of proteins identified within each, the GRAVY score was compared (Figure 5D). There were no clear differences in the distribution of GRAVY scores for each SPM, suggesting hydropathy of native proteins was not a factor. Statistical analysis comparing protein abundances between SPMs found WP to have higher recoveries of most proteins and no clear shift in GRAVY scores (FDR < 0.05; supplementary). The PF treatment exhibited the second greatest number of protein identifications and average protein abundances with a total of 581 proteins which were significantly higher and 900 significantly lower when compared to WP. Average GRAVY scores were not very different between proteins that were significantly higher and lower when compared to WP (−0.415 & −0.380 respectively).

With respect to the protein discs, protein identifications and reproducibility across replicates were consistent, although being lower than the PF and WP SPMs. Surprisingly, there were relatively few proteins identified in common between the two protein disc SPMs (Figure 5C). The PF Disc and WP Disc with modified-bligh dyer extraction had fewer proteins than PF and WP, but performed similarly to PF in terms of recovery of proteins that were identified. 19% of proteins had significantly lower abundances for the PF Disc while, PF had 17 and 15% which reflects relatively good recovery of many hundred proteins for each SPM. A comparison between the WP disc and PF disc found 594 proteins which were significantly higher in the PF Disc and 626 that were significantly lower. Considering the PF DPS was used to create the PF Disc, this DPS method offers good performance relative to the WP disc.

### 3.4 Lipidomics

**FIGURE 6:**
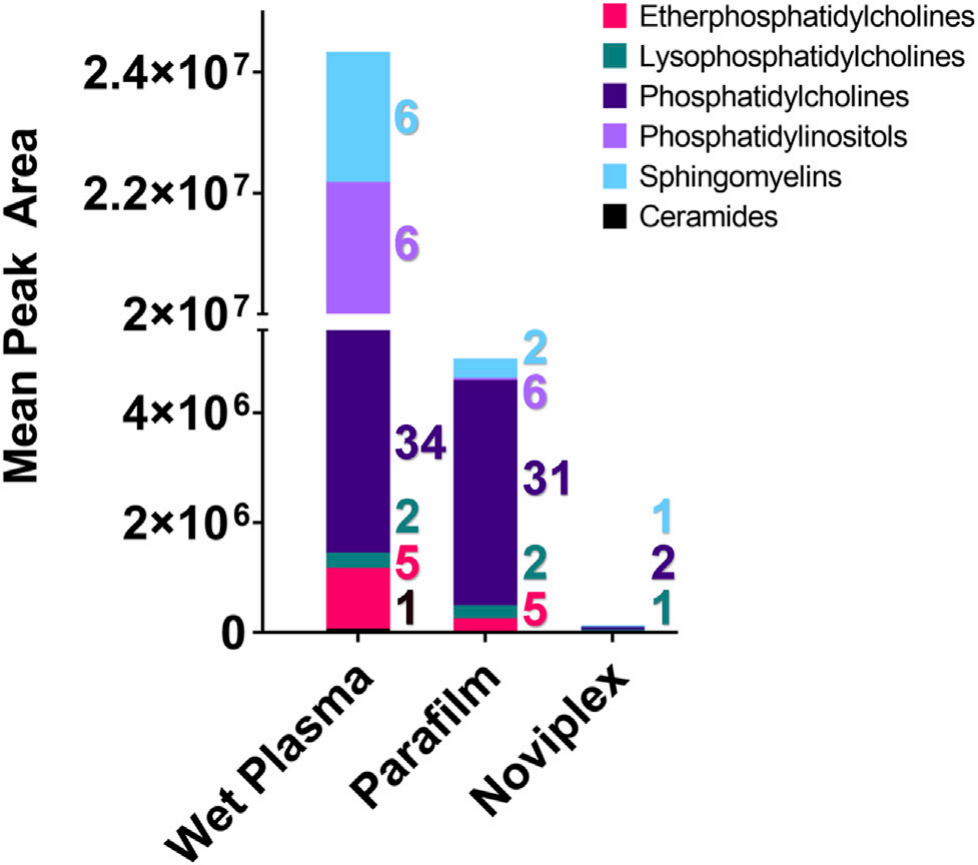
The summed average area of chromatographic peaks (Gaussian smoothed) for different lipid classes identified within each Sample processing method (WP, PF, & NP). Each coloured segment of the stacked bar graph represents the mean chromatographic area for the respective lipid class. The colour coded numbers to the right of each bar represent the number of lipids that were detected in each category. Abbreviation: Cer, Ceramide; EPC, Ether Phosphatidylcholine; LPC, Lysophosphatidylcholine; PC, Phosphatidylcholine; PI, Phosphatidylinositol; SM, Sphingomyelin.

The composition and quantity of lipids detected in each treatment differed quite a bit (Figure 6). In terms of quantity, the WP, PF, and NP mostly contained phosphatidylinositol, phosphatidylcholine, and lysophosphatidylcholine, respectively. Although % recovery was not calculated within the study, individual lipid abundances are relatively similar between the PF DPSs and the WP except for the phosphatidylcholines which were tended to be higher in the PF DPSs (Table 1).

**TABLE 1:** Relative fold-change (PF/WP) and significance of lipids recovered from Parafilm™ (PF) dried plasma spots compared to wet plasma (WP). Significance testing was conducted using a One-Way-ANOVA, Tukey’s post-hoc with Bonferroni-Hochberg FDR correction of p-values (significance at FDR < 0.05).

Compound name	Fold change	FDR-Adjusted p-Value
LPC 16:0/0:0	0.024782	2.12E-06
PC 16:1_18:1	0.082263	1.12E-05
PC 16:0_18:1	0.25035	7.72E-05
PC 16:0_16:1	0.27086	0.000253
PC 16:0_20:4	0.30445	0.00043
PC 18:1_18:2	0.31187	0.000574
PC 16:0_18:2	0.34537	0.000574
PC 16:0_20:3	0.35238	0.001322
PC 16:0_22:6	0.36828	0.005572
PC 16:0_20:5	0.53819	0.005699
PC 18:1_22:6	0.61048	0.008124
SM d37:3	1.4839	0.015681
SM d42:3	1.5429	0.015681
SM d40:2	1.5461	0.028347
SM d42:2	1.5689	0.028347
PI 16:0_20:4	1.7118	0.041039
SM d43:4	1.7734	0.04257
PC 18:0_20:3	2.0052	0.043251
PI 16:0_22:6	2.559	0.045346
Cer_NDS d16:0_26:2	3.5639	0.045346

With respect to the number of lipid classes and unique lipids detected in each group, the PF and WP treatments performed similarly. We detected the most unique lipids (60) and number of lipid classes (6) within the WP, with a single ceramide being detected only within this sample SPM. The PF samples contained 44 unique lipids from five lipid classes, while the NP samples contained only four unique lipids from three lipid classes (Figure 5).

There was a significant difference found between the mean peak area (relative abundance) of several lipids recovered using PF DPSs compared to wet plasma, with peak areas typically being highest for WP (Table 1). There was a significant difference found between 20 lipids in the WP and PF groups (FDR < 0.05); 10 phosphatidylcholines (PCs) and 1 lysophosphatidylcholine (LPC), were higher in the PF treatment; whereas 5 sphingomyelins (SMs), 2 Phosphatidylinositols (PIs), 1 phosphatidylcholine (PC) and 1 ceramide (CM) were higher in the WP treatment. With respect to the NP, LPC 16:0/0:0 and PC 16:0_18:1 were significantly higher than WP while PC 16:0_18:2 was significantly lower. The abundance of SM d42:2 was not significantly different compared to WP.

### 3.5 Amino Acid Results

**FIGURE 7:**
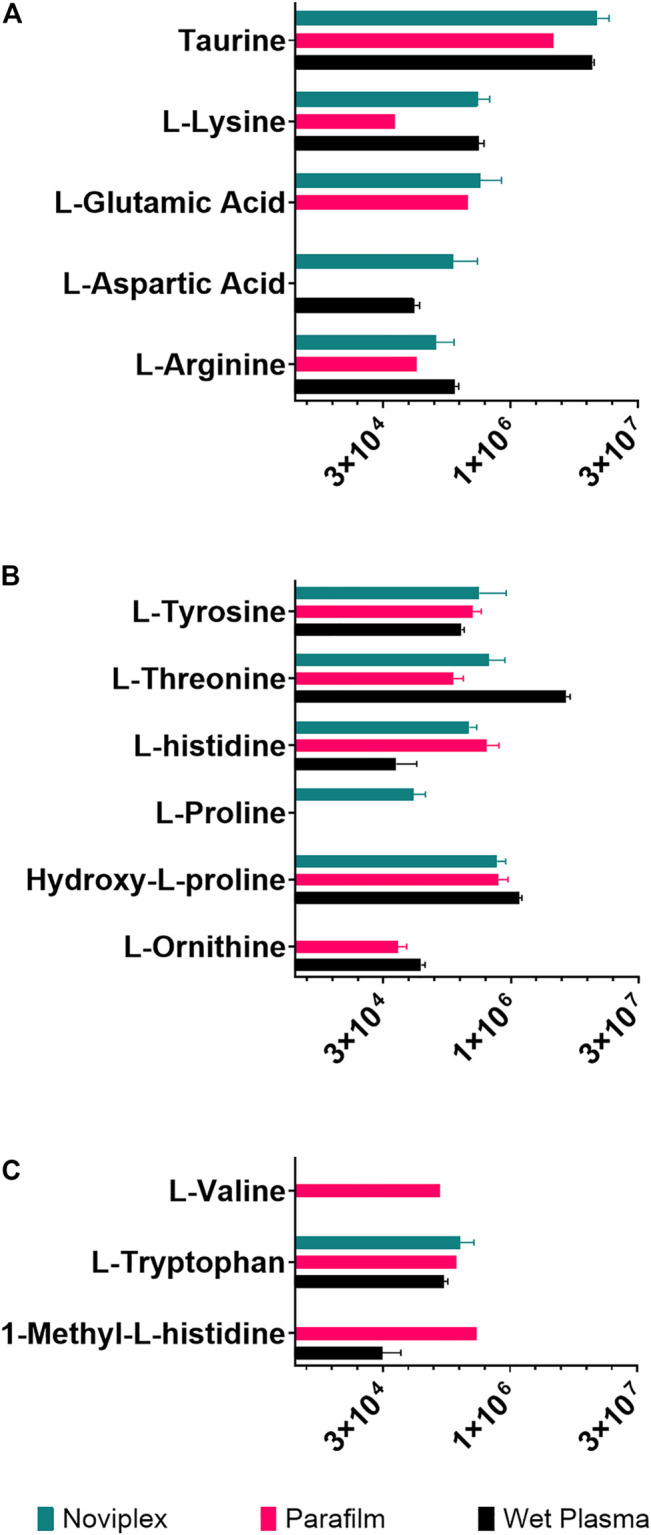
The summed average area of chromatographic peaks (Gaussian smoothed) for amino acids grouped by hydropathy **(A)** Hydrophilic **(B)** Neutral **(C)** Hydrophobic for each sample processing method. The error bars indicate standard deviation.

The recovery of amino acids was similar for the DPSs compared to the WP (Figure 7). A total of 11 amino acids were identified in the NP DPS, 13 in the PF DPS and 9 for the WP. Statistical analysis uncovered more subtle differences between the two DPS methods (Table 2). Overall the NP DPSs performed better than the PF, with 9 amino acids found to be significantly higher in abundance while only 3 were found to be significantly lower than the PF group. Additionally, the PF DPSs tended to have lower peak areas and were more variable (higher standard deviation) for all analytes except L-Histidine and Hydroxy-L-proline (Supplementary Table S3). We did not detect 1-Methyl-L-Histidine, L-Ornithine and L-Valine in the NP DPSs, whereas L-Aspartic acid was not detected in the PF DPSs.

**TABLE 2:** Relative fold-change (NP/PF) and significance of amino acids recovered from Noviplex™ (NP) cards compared to Parafilm™ (PF) dried blood/plasma spots. Significance testing was conducted using a One-Way-ANOVA, Tukey’s post-hoc with Bonferroni-Hochberg FDR correction of p-values (significance at FDR < 0.05).

Compound name	Fold change	FDR-Adjusted p-Value
L-Proline	8.6646	1.951E-15
L-Arginine	4.774	7.1585E-08
1-Methyl-L-histidine	0.038405	5.1078E-07
L-Aspartic Acid	68.087	5.1078E-07
L-Valine	0.031054	9.2703E-07
L-Glutamic Acid	4.0491	9.2703E-07
L-Ornithine	0.027394	1.4012E-06
L-Lysine	26.146	0.000016606
L-Threonine	6.9272	0.00065723
L-Tryptophan	3.0784	0.00065723
Hydroxy-L-proline	2.5905	0.00088258
Taurine	8.521	0.00088258

## 4 Discussion

Blood and blood plasma are attractive biofluids for non-lethal sampling in biomonitoring programs. Until now, there was no openly available information on the effect of sampling blood on the healing and survival of fish, which has likely restricted the use of blood and plasma in field research studies. Some valuable data has already been generated from blood and blood plasma by many researchers in the past, but none have explicitly reported mortality and healing of fish following phlebotomy (Lawrence et al., 2020; Cook et al., 2011; 24–28). We thought that an established method with data on stress and survival would enable researchers to monitor the health of threatened and endangered fish species without concern for population impacts. We present data which validates blood-sampling from the caudal vasculature as a truly non-lethal method. In addition, we present data on the utility of dried plasma spots for protein and metabolite analyses. We tested specialized plasma preparation cards because they offer an attractive option for separation of plasma from whole blood samples, without the need for centrifugation or immediate cold storage, and compared their performance to the same volumes of dried and wet plasma.

### 4.1 Non-Lethal Blood Sampling

#### 4.1.1 Non-Lethal Blood Sampling in the Laboratory

In order to assess the potential lethality of blood sampling from fish, we monitored survival and healing for several weeks after sampling blood from the caudal vasculature in the laboratory and in the field. The laboratory study evaluated several post-sampling treatments to the puncture site in order to test whether any would improve healing and survival. It was found that the pressure-only treatment resulted in the highest survival rate (100%; Figure 3) at 4-weeks post sampling which suggests that minimal intervention is better for healing at the puncture site. Minimizing handling times and air exposure is known to be important for reducing stress, ultimately affecting fish welfare (Cook et al., 2015; Lawrence et al., 2018).

We observed that applying Betadine™ and liquid bandage treatments was detrimental to fish health, considering that survival post-sampling was less than 90% and wounds persisted for weeks, which may have prolonged their suffering (Supplementary Table S1). The appearance of redness along the abdomen of the fish within these treatments also suggests that the natural mucosal barrier which protects fish from infection was disturbed (Gomez et al., 2013). Relatively few topical antiseptics are recommended for use on fish because they can adversely affect the skin and mucosa (Mulcahy, 2003). For example, a previous study found a 10% iodopovidone (Betadine™) application did not improve healing and caused some skin irritation in rainbow trout following surgical implantation of a transmitter (Wagner et al., 1999). Within our own study, application of the fish’s own mucous resulted in a 95% survival rate, which suggests even this intervention does not appear to improve fish condition and survival in this study. Thus, it is likely that minimizing handling times with no post treatment to the wound, aside from pressure to help with clotting, is the best possible method for sampling blood from the caudal vasculature of fish.

#### 4.1.2 Non-Lethal Blood Sampling in the Field

As a follow-up to the laboratory pilot, caudal blood sampling was tested in the field to evaluate whether more complex, natural, freshwaters would impact the survival and healing of rainbow trout. Daily visual inspection of fish in their cages during the 2-weeks monitoring period indicated that the fish healed quickly with no infection, exhibiting a 95% survival rate with no significant difference in the proportion of mortality compared to fish that were caged without blood sampling (Supplementary Figure S5). These findings strongly support blood sampling as a viable non-lethal option for wild fish. Throughout the 2-weeks monitoring period, only one mortality was observed in a fish whose blood was sampled.

The laboratory aquatics facility offers a very controlled environment with 12°C de-chlorinated water maintained by a flow-through system, with 5-tank turnovers every 24 h (Sprague, 1973). However, the natural defense system(s) of the rainbow trout were clearly sufficient to protect them from potential bacterial threats in the creek. Our data and observations support the use of blood sampling via caudal puncture with 30 s of pressure as a non-lethal technique for fish in both the laboratory and the field with a 95–100% survival rate.

### 4.2 Dried Plasma Spots

We tested the efficacy of using dried plasma spots (DPSs) compared to wet plasma, with a card system (NP) that did not require centrifugation, and with plasma that was dried on parafilm (PF), where both spots did not require subsequent cold storage for preservation. We employed an untargeted MS/MS data acquisition with a targeted bioinformatic approach for lipids, amino acids, and proteins to compare the performance of the two DPS techniques. We found that the success of these two DPS techniques varied depending on the analysis conducted. Wet plasma generally performed better than DPS for lipid and proteomic analyses with greater abundances and number of uniquely identified analytes. The PF approach appeared to be the best option for DPS as there was greater abundances and more identifications of lipids and proteins than the NP DPSs. The NP spot cards performed very poorly for the lipid and protein analyses, but did demonstrate success for amino acids over the PF and WP sample preparation methods.

#### 4.2.1 Proteomics

There was an average of 900, 757, 644, and 621 protein identifications made for the WP, PF, WP Disc, and PF Disc sample preparation methods (SPMs), respectively, which offers many possibilities for biomarker discovery and analysis. When compared to WP, the PF, PF Disc, and WP Disc SPMs still performed relatively well considering dried plasma and blood spots generally have lower recoveries and identifications of proteins relative to regular plasma samples (Chambers et al., 2013; Nakajima et al., 2020). In order to estimate protein recoveries for SPMs, we calculated the fold change of the SPMs relative to WP. The DPS methods exhibited significantly lower protein abundances compared to the WP for 19% of proteins identified within the PF Disc, 17% for PF (17%) and 15% for the WP Disc. Recovery of proteins is less important for biomarker analysis if relative measurements can be used to determine differences in protein abundance. DBSs can be used to enrich specific classes of proteins based on their physical properties. For example, Chambers et al. (2013) used Cytiva Whatman 903 protein saver cards and found a significantly higher recovery of hydrophobic proteins compared to wet plasma, as determined by the grand average of hydropathy (GRAVY) (Kyte and Doolittle, 1982). Within our own study, we did not find any clear differences in the hydropathy of proteins across SPMs. Over 90% of proteins detected within the current dataset had negative GRAVY values suggesting that both proteomics sample preparation methods (i.e., Bligh-Dyer extraction or dissolution in AB buffer) select a similar range of mostly hydrophilic proteins regardless of the SPM used. Hydrophobic, native proteins tend to occupy cellular membranes and are not recovered well without specialized sample extraction protocols such as the use of a detergent (Ludwig et al., 2018; Nakajima et al., 2020). It was expected that the PF DPS may affect the abundances of hydrophobic proteins considering it is a polymer primarily composed of paraffin waxes and polyolefins which create a non-polar support material, but there were few hydrophobic proteins identified within the dataset (Miller, 2012). Nakajima et al., 2020 reported a relatively simple treatment of DBSs extracts with sodium carbonate that allowed substantial removal of high abundance hydrophilic proteins which enriched hydrophobic proteins from DBSs. Depending on the context, larger amounts of time and reasource can be invested in the lab following sample collection for additional sample processing and enrichment of specific classes of proteins. Thus, it is entirely possible that DBS card use will become more popular for use in environmental effects monitoring considering the convenience and performance in recovery of different analytes. A particularly noteworthy SPM was the PF disc because although it did not perform the best when compared to the other SPMs, it is still worthwhile as it also permits analysis of lipids and amino acids from a single 30 ul blood sample. Thus, depending on the objectives of a study, extracting biomolecules from a DPS using the modified-Bligh-Dyer may be a better option considering it allows a multi-Omics analysis at the cost of some performance in protein recovery and sensitivity.

It is also clear that some types of support materials perform better than others when it comes to proteomics analysis. For example, cellulose based blood spot cards have demonstrated the success for proteomics in terms of protein identifications and recoveries when compared to WP (Chambers et al., 2013; Nakajima et al., 2020). Within our own study, the NP DPSs did not perform well for proteomics analysis, and no proteins were identified in any replicate (n = 10). A multitude of possibilities could explain the reason behind this; it is possible that the NP card is better suited towards smaller, polar classes of metabolites as determined in the amino acid analysis. It was hypothesized that size exclusion was the reason for no proteins being detected in NP samples which could have occurred during the initial filtration step that removed blood cells from the plasma. However, this cannot be confirmed because we do not know the pore size of the filter membrane or the nature of the materials used in the NP cards due to manufacturer trade secret. Regardless, a review of the literature demonstrates the great success for proteomics analysis of DBSs on cellulose-based/paper collection devices, and a DBS system that was more specialized for protein analysis may have improved the outcome (Chambers et al., 2013; Björkesten et al., 2017; Rosting et al., 2018; Nakajima et al., 2020).

#### 4.2.2 Lipidomics

We observed the most lipid identifications and relative abundances in the wet plasma, except for the phosphatidylcholine (PC) lipid class, which were generally higher for the PF DPSs (Figure 6). PF is a non-polar support material, which could explain the higher recoveries observed for phosphatidylcholines in the PF DPSs (Miller, 2012). This finding contrasts with some results in a study conducted by Aristizabal-Henao et al. (2016) wherein they compared the performance of Dried blood spots (DBSs) on Whatman cellulose chromatography paper and found recovery of phosphatidylcholines, lysophosphatidylcholines and phosphatidylethanolamines was the same as whole blood following a 24 h incubation period with extraction solvent (Aristizabal Henao et al., 2016). It was found that lengthening the extraction period to 24 h in combination with homogenization of the cellulose, paper disks caused large improvements to analyte recovery (Aristizabal Henao et al., 2016). The plasma collection disk materials and additives used in the NP card are proprietary, and therefore we are unable to explore why they did not work particularly well for lipid analysis. However, homogenization would have been difficult because the material is very hard, and we were reluctant to test it in case we damaged our equipment. Generally, many lipid analyses targeting lipids of clinical importance (i.e., triglycerides, cholesterol, lipoproteins etc.) have had reduced recovery and sensitivity from DBSs when compared with wet plasma (Lakshmy et al., 2012; Samuelsson et al., 2015; Corso et al., 2016). A study by Snowden et al. (2020) identified 71% of the lipids found in wet plasma in dried blood spots, which is similar to our results with the PF DPSs in the present study. Additionally, lipid concentrations could be corrected with the use of internal standards, as was done by Snowden et al. (2020). Overall, lipid analysis of dried blood and plasma spots shows promise and could be used with field blood samples, without requiring large plasma volumes or cold storage. It is likely that with method optimization for extraction, use of internal standards, and improvements in spot card technology, dried plasma and blood spotting will be a viable method for lipid biomarker analysis in the future.

#### 4.2.3 Amino Acids

Generally, amino acid analyses using dried blood spots has shown success with relatively high recoveries and numbers of identifications when compared to WP (Wagner et al., 2016; Bloom et al., 2019; Giordano et al., 2019; van Vliet et al., 2020). Bloom et al., 2019 reported recoveries of spiked amino acid standards between 85 and 99%, with only slightly lower precision relative to plasma which validates the use of dried blood spots for analysis of many amino acids (Bloom et al., 2019). In the present study, the DPSs had similar recoveries of amino acids relative to the WP as well, having higher numbers of identifications than WP being 13 for NP, 11 for PF and 9 for WP. PF spots performed similarly to the NP, with more identifications of amino acids (Figure 7A-C; 13 compared to 9) but generally having lower abundances except for L-valine, 1-methyl-histidine, and L-Ornithine. These results suggest that drying plasma improves the recovery of nonpolar metabolites using a liquid-liquid extraction technique (Figure 7). We also observed that the recovery of the polar amino acids (and derivatives) were significantly higher for the NP spots including aspartic acid, lysine, glutamic acid and taurine while recoveries of the most hydrophobic amino acids were significantly higher for the PF spots, including valine and 1-methyl-L-histidine. Additionally, with respect to the neutral amino acids, the relative recoveries were similar. It is possible that the NP card collection spot material is better suited to small, polar metabolites, and suggests that one way to improve DPSs in the future could be to develop specialized surface supports to capture specific classes of metabolites.

#### 4.3 Summary and Conclusion

The results of the present study demonstrate that blood can be sampled non-lethally from fish and we recommend that these procedures be used more frequently to monitor wild fish without euthanization. More specifically, sampling blood from the caudal vasculature with applied pressure appears to be a robust, non-lethal sampling method. Especially when sampling blood from threatened species, we hope that the results of our study will enable researchers, fisheries, & other stakeholders to ethically assess fish health and protect fish populations. Many government-run biomonitoring and environmental effects monitoring programs around the world currently sacrifice thousands of fish each year to determine if the fish are healthy, and it is hypocritical and unnecessary to do so in the name of conservation. The use of DBSs and DPSs for processing and storage of whole blood samples would benefit sampling in remote areas and overcome logistical constraints involved with traditional haematological analyses. As well, our data provide evidence that a relatively inexpensive material, parafilm, can be effective for drying small volumes of plasma, which can then provide high quality biomarkers of health from subsequent proteomic, lipidomic and amino acid analyses. We would recommend using the parafilm DPS in conjunction with the modified Bligh-Dyer extraction method because it offers a good compromise; allowing good recoveries and identifications for the three classes of biomolecules tested from a single 30 µl draw volume of whole blood (resulting in 15 µl of dried plasma, in our case). Unfortunately, the NP DBS card performed poorly for lipidomics and proteomics, but was the best technique for analysis of amino acids. It is likely that improvements in DBS card technology and use of metabolite class specific cards when blood volume is not limited will make DBSs an attractive option for environmental effects monitoring in the coming years.

## Data Availability

The original contributions presented in the study are included in the article/Supplementary Material, further inquiries can be directed to the corresponding author.
