# Supplementary material for: Non-Lethal Blood Sampling of Fish in the lab and Field With Methods for Dried Blood Plasma Spot Omic Analyses
Source: Front Genet. 2022 Mar 24;13:795348. doi: 10.3389/fgene.2022.795348 (PMC8988233; doi:10.3389/fgene.2022.795348)
Supplement: Supplementary file 3 [file Table1.DOCX]

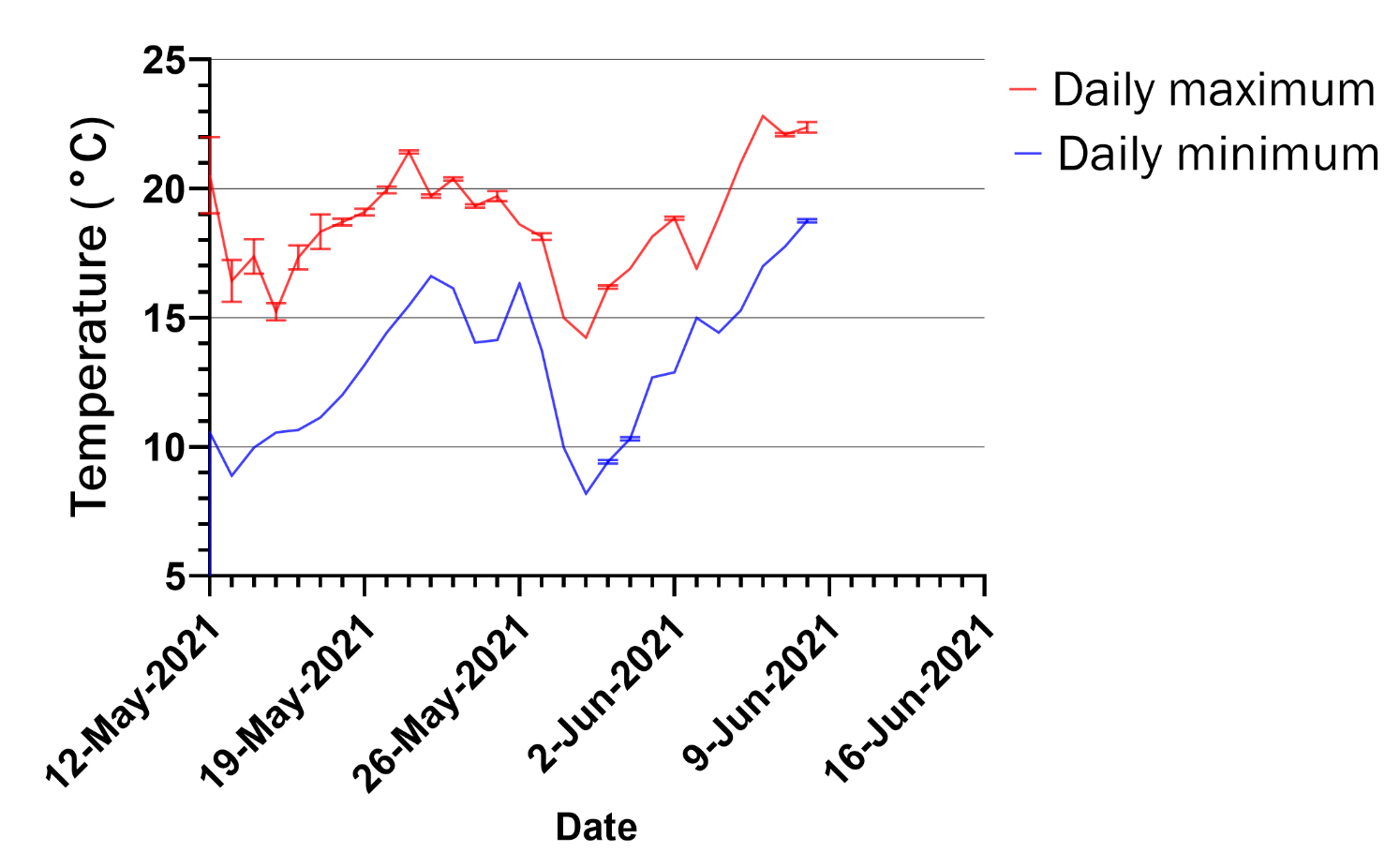


**Supplementary Figure 1.** Daily minimum and maximum temperatures recorded in Oshawa Creek from May 12 to June 9, 2021. Temperatures are averaged across data collected from each cage, error bars represent standard deviation.

**
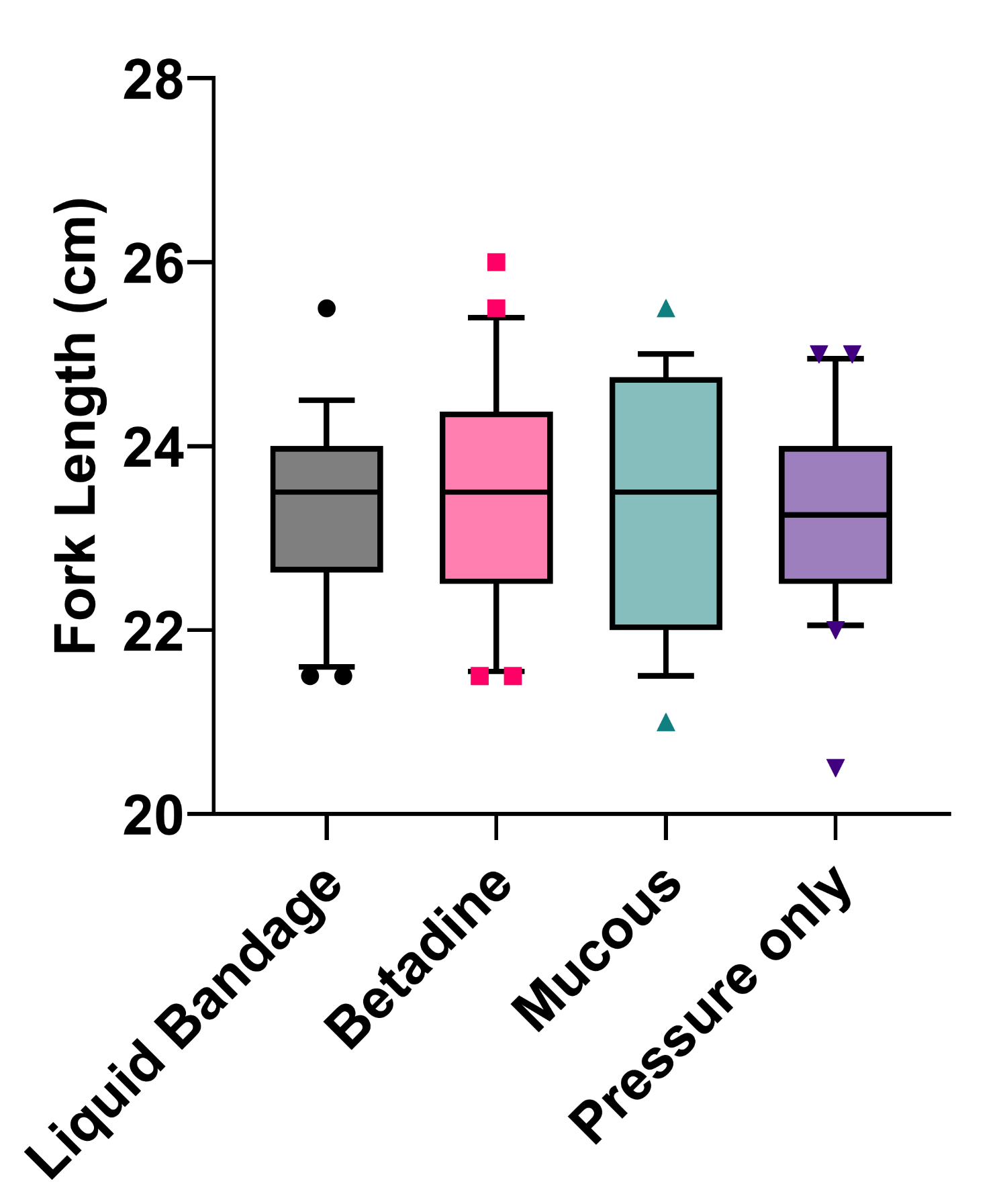
**

**Supplementary Figure 2.** Fork Length (cm) of Rainbow Trout used in the Laboratory Blood Sampling Experiment. Length of animals is grouped by the post-sampling treatment to the puncture site (Betadine, Liquid Bandage, Fish Mucous or Pressure-Only). Box & whisker plot illustrates the median, 10-90 percentiles and symbols represent data points outside that range.

**
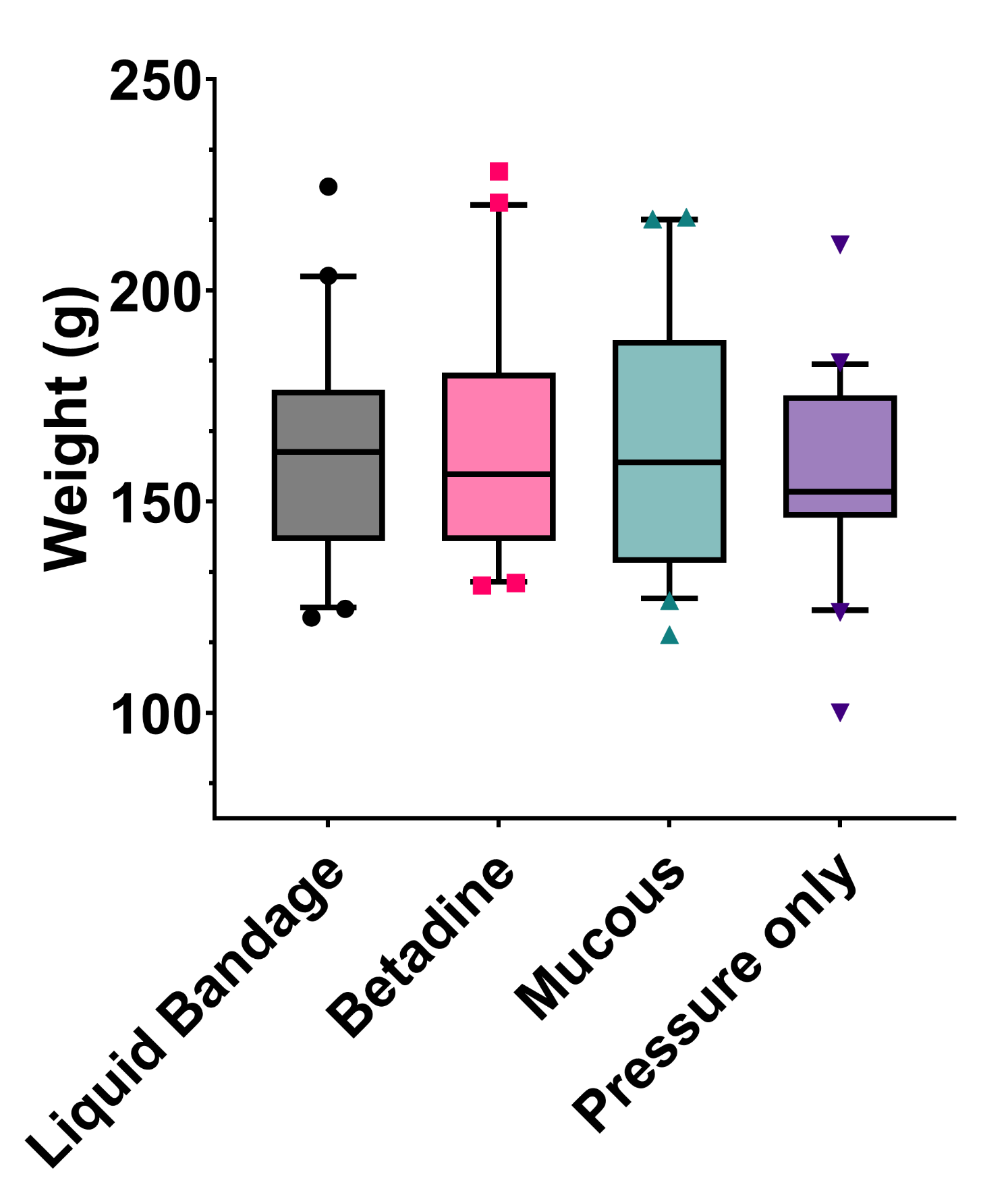
**

**Supplementary Figure 3.** Weight in grams of Rainbow Trout used in the Laboratory Blood Sampling Experiment. Weight of animals is grouped by the post-sampling treatment to the puncture site (Betadine, Liquid Bandage, Fish Mucous or Pressure-Only). Box & whisker plot illustrates the median, 10-90 percentiles and symbols represent data points outside that range. **
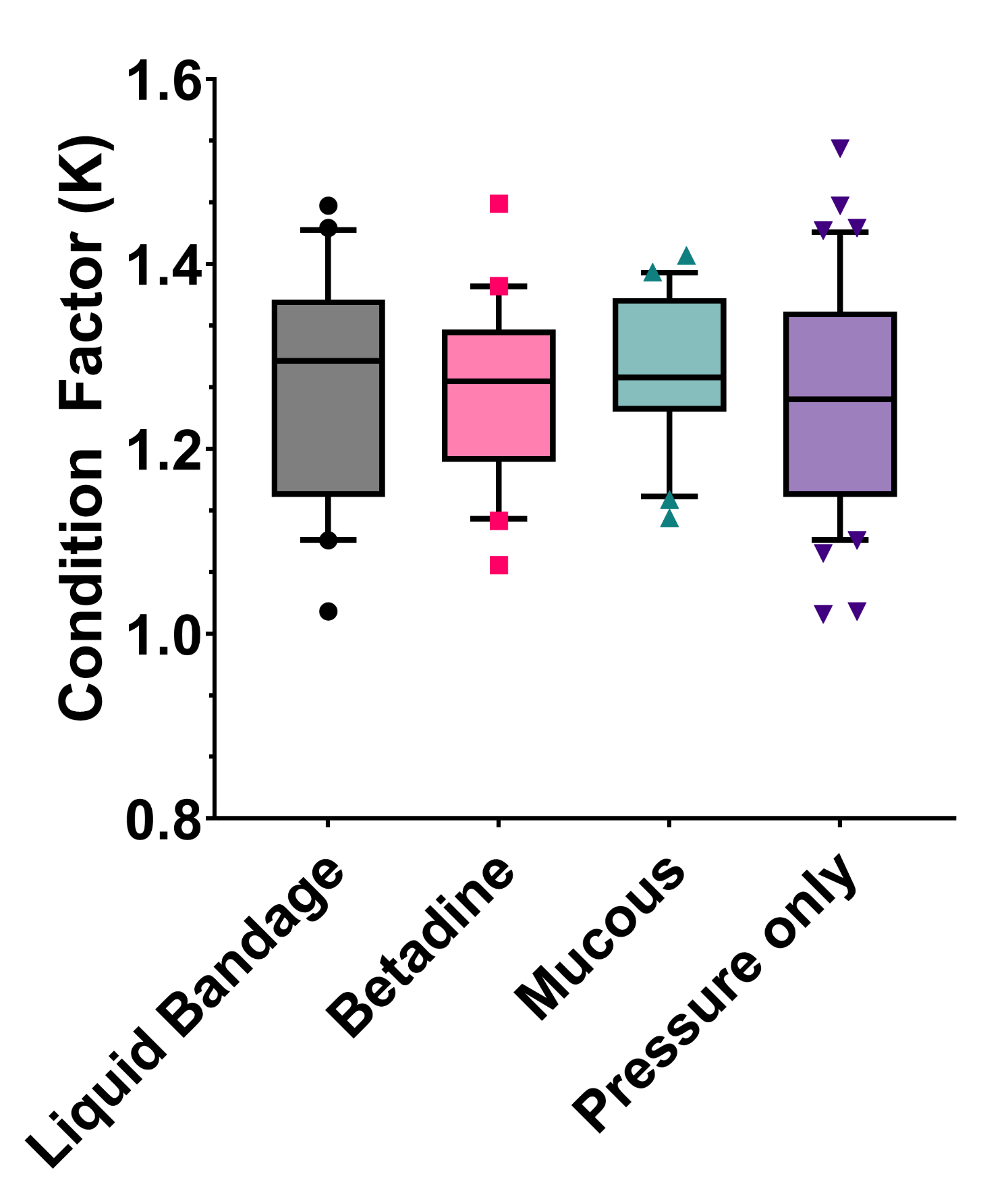
Supplementary Figure 4.** Condition factor of Rainbow Trout used in the Laboratory Blood Sampling Experiment. Condition factor of animals is grouped by the post-sampling treatment to the puncture site (Betadine, Liquid Bandage, Fish Mucous or Pressure-Only). Box & whisker plot illustrates the median, 10-90 percentiles and symbols represent data points outside that range.

| Betadine | | | | |
| --- | --- | --- | --- | --- |
|  | Week 1 | Week 2 | Week 3 | Week 4 |
| Healed | 15 | 16 | 14 | 15 |
| Swollen/red tail | 2 | 1 | 1 |  |
| Swollen/red abdomen | 2 | 1 |  |  |
| Both |  | 1 |  |  |
| Mortality | 1 | 2 | 5 | 5 |
|  |  |  |  |  |
| Liquid bandage | | | | |
|  | Week 1 | Week 2 | Week 3 | Week 4 |
| Healed | 15 | 16 | 14 | 15 |
| Swollen/red tail | 2 | 1 | 1 |  |
| Swollen/red abdomen | 2 | 1 |  |  |
| Both |  | 1 |  |  |
| Mortality | 1 | 2 | 5 | 5 |
|  |  |  |  |  |
| Mucous Swab | | | | |
|  | Week 1 | Week 2 | Week 3 | Week 4 |
| Healed | 15 | 155 | 16 | 19 |
| Swollen/red tail | 1 | 3 | 2 |  |
| Swollen/red abdomen | 4 | 2 | 1 |  |
| Both |  |  |  |  |
| Mortality |  |  | 1 | 1 |
|  |  |  |  |  |
| Pressure Only | | | | |
|  | Week 1 | Week 2 | Week 3 | Week 4 |
| Healed | 18 | 18 | 20 | 20 |
| Swollen/red tail | 1 | 1 |  |  |
| Swollen/red abdomen |  |  |  |  |
| Both | 1 | 1 |  |  |
| Mortality |  |  |  |  |

**Supplementary Table 3.** Healing of Rainbow Trout in the Laboratory Monitored Weekly After Sampling Blood from the Caudal Vasculature. Healing of animals is grouped by the post-sampling treatment to the puncture site (Betadine, Liquid Bandage, Fish Mucous or Pressure-Only).

**Supplementary Table 2**. Lipids quantified in Wet Plasma and Dried Plasma Spots (Parafilm and Noviplex^TM^). STDEV (Standard Deviation); WP (Wet Plasma); PF (Parafilm); NP (Noviplex^TM^)

| Lipid | WP Peak Area | WP STDEV | PF Peak Area | PF STDEV | NP Peak Area | NP STDEV |
| --- | --- | --- | --- | --- | --- | --- |
| Cer_NDS d16:0_26:2 | 68309.11 | 51603.20 |  |  |  |  |
| EtherPC 16:0e_20:4 | 102234.78 | 41068.53 | 31314.6923 | 33590.6802 |  |  |
| EtherPC 16:0e_20:5 | 100360.00 | 34086.26 | 19642.5 | 14997.5462 |  |  |
| EtherPC 16:0e_22:6 | 442751.00 | 165805.79 | 417921.116 | 628048.559 |  |  |
| EtherPC 18:1e_20:4 | 119786.56 | 51637.56 | 21142.4 | 11839.1335 |  |  |
| EtherPC 18:1e_22:6 | 345727.00 | 152404.55 | 391815.915 | 606374.699 |  |  |
| LPC 16:0/0:0 | 72501.67 | 28706.76 | 213386.5 | 106804.58 | 49992.1667 | 17923.6524 |
| LPC 22:6/0:0 | 205653.44 | 77053.56 | 22954.9 | 12411.1457 |  |  |
| PC 14:0_16:1 | 85718.67 | 36653.66 | 16834.1111 | 9080.58522 |  |  |
| PC 14:0_20:5 | 88229.00 | 40204.08 | 822310.61 | 1342351.97 |  |  |
| PC 14:0_22:6 | 274134.00 | 86170.68 | 35816.4 | 24535.3046 |  |  |
| PC 16:0_16:1 | 487774.33 | 125078.77 | 247114.6 | 253121.161 |  |  |
| PC 16:0_18:1 | 1701217.00 | 445567.57 | 632311.9 | 330646.087 | 47122.6667 | 8156.07052 |
| PC 16:0_18:2 | 994245.67 | 357195.19 | 180082.851 | 192281.258 | 8281.16667 | 1142.86454 |
| PC 16:0_20:3 | 421962.00 | 153845.24 | 104619.4 | 86868.9292 |  |  |
| PC 16:0_20:4 | 400422.00 | 97052.69 | 320549.872 | 388293.003 |  |  |
| PC 16:0_20:5 | 1917123.89 | 449930.12 | 397502.9 | 295288.001 |  |  |
| PC 16:0_22:4 | 101551.22 | 35250.72 | 239211.209 | 401746.85 |  |  |
| PC 16:0_22:5 | 563001.00 | 129484.42 | 125254 | 97848.7269 |  |  |
| PC 16:0_22:6 | 3053271.89 | 675028.39 | 236490.571 | 406143.721 |  |  |
| PC 16:1_18:1 | 119207.60 | 52722.96 | 42653.6667 | 26232.9405 |  |  |
| PC 16:1_18:2 | 97892.89 | 67300.95 | 56780.3171 | 59672.0385 |  |  |
| PC 16:1_22:6 | 730572.11 | 221230.24 | 79538.4 | 68353.3001 |  |  |
| PC 18:0_18:1 | 253850.89 | 155648.02 | 93348.3333 | 73436.7835 |  |  |
| PC 18:0_18:2 | 265774.89 | 138316.10 | 47531.5714 | 24522.3076 |  |  |
| PC 18:0_20:3 | 82876.44 | 46264.18 |  |  |  |  |
| PC 18:0_22:5 | 133225.11 | 68840.26 |  |  |  |  |
| PC 18:0_22:6 | 819243.44 | 303531.75 | 107301.4 | 83692.2032 |  |  |
| PC 18:1_18:1 | 532682.78 | 221634.06 | 135165.75 | 70114.4682 |  |  |
| PC 18:1_18:2 | 414077.33 | 146253.01 | 133481.14 | 161350.231 |  |  |
| PC 18:1_20:1 | 114268.22 | 76252.68 | 21052.25 | 9804.93333 |  |  |
| PC 18:1_20:5 | 605626.89 | 235411.55 | 130266.286 | 163501.417 |  |  |
| PC 18:1_22:6 | 1714553.22 | 483740.96 | 305792.9 | 233843.582 |  |  |
| PC 18:2_20:5 | 269910.89 | 104268.70 | 48261.1389 | 53782.7609 |  |  |
| PC 18:2_22:6 | 621969.44 | 172886.00 | 92936.9 | 82326.9278 |  |  |
| PC 18:3_22:6 | 97178.78 | 75611.15 | 35041.3333 | 32840.1062 |  |  |
| PC 20:1_18:2 | 154867.33 | 75640.66 | 40851.2857 | 24768.2029 |  |  |
| PC 20:1_20:5 | 203159.63 | 140340.02 | 88533.7179 | 114049.677 |  |  |
| PC 20:1_22:6 | 309209.67 | 156693.09 |  |  |  |  |
| PC 20:5_22:6 | 260415.67 | 109050.28 | 21401.1111 | 20120.3095 |  |  |
| PC 22:6_22:6 | 473216.33 | 127987.88 | 453862.024 | 564547.699 |  |  |
| PC 32:2 | 29406.13 | 8876.36 | 9235.33333 | 1590.69335 |  |  |
| PI 16:0_20:4 | 282880.56 | 133682.56 | 382572.174 | 664094.528 |  |  |
| PI 16:0_22:6 | 460425.56 | 235868.03 | 20011.3333 | 15976.0118 |  |  |
| PI 18:0_20:4 | 934677.67 | 408086.44 |  |  |  |  |
| PI 18:0_22:6 | 194871.00 | 78719.00 |  |  |  |  |
| PI 18:1_20:4 | 303672.33 | 141751.88 |  |  |  |  |
| PI 18:1_22:6 | 158767.89 | 75106.22 |  |  |  |  |
| SM d32:1 | 280684.67 | 125785.02 | 59949.35 | 72218.3569 |  |  |
| SM d37:3 | 101260.89 | 40239.70 | 11718 | 210.717821 |  |  |
| SM d40:2 | 161814.33 | 85472.66 | 110627.625 | 95165.986 |  |  |
| SM d42:2 | 1152953.67 | 521227.59 | 162104.6 | 83742.5864 | 21610 | 4020.16957 |
| SM d42:3 | 201788.22 | 113045.55 |  |  |  |  |
| SM d43:4 | 254114.44 | 157833.60 |  |  |  |  |

| Amino Acid | WP Peak Area | WP ST DEV | PF Peak Area | PF STDEV | NP Peak Area | NP STDEV |
| --- | --- | --- | --- | --- | --- | --- |
| Threonine-13C4 15N | 632822.78 | 1223449.36 | 1419944.62 | 754813.03 | 18706411.73 | 10126486.83 |
| Taurine | 432455.24 | 798775.89 | 3356615.19 | 1201326.46 | 11125985.46 | 4264380.52 |
| Hydroxy-L-proline | 64222.25 | 82960.25 | 747959.18 | 217467.47 | 717883.33 | 191835.20 |
| L-Threonine | 98739.05 | 131536.88 | 220481.68 | 66837.75 | 577145.19 | 313802.90 |
| L-Tryptophan | 38114.03 | 50588.71 | 244367.74 | 63476.56 | 271382.33 | 121015.08 |
| Tryptophan- 13C11 15N2 | 471681.46 | 860554.68 | 9912406.58 | 3590691.85 | 27686756.51 | 16864010.92 |
| L-Lysine | 36296.18 | 48441.10 | 45386.87 | 11069.29 | 433526.07 | 162172.41 |
| Tyrosine-13C9 15N | 321285.70 | 619318.19 | 4978108.10 | 2445379.08 | 30506392.57 | 25608014.44 |
| Glutamic acid-13C5 15N |  |  | 162876.56 | 72001.18 | 11407822.05 | 10664712.92 |
| Valine-13C5 15N |  |  | 1459043.95 | 520066.89 | 4022642.66 | 1369954.87 |
| L-Ornithine |  |  | 48976.48 | 12852.80 |  |  |
| 1-Methyl-L-histidine |  |  | 423518.98 | 180579.35 |  |  |
| Isoleucine-13C6 15N |  |  | 3582240.32 | 1739973.14 | 16624444.22 | 12595897.47 |
| L-Valine |  |  | 156388.09 | 48744.57 |  |  |
| L-Tyrosine |  |  | 373731.86 | 92867.19 | 443930.17 | 477170.99 |
| L-Histidine |  |  | 548893.31 | 208746.70 | 336328.09 | 78454.07 |
| L-Arginine |  |  | 81076.81 | 15742.63 | 138504.00 | 87673.10 |
| L-Glutamic Acid |  |  | 328876.78 | 57710.94 | 458962.97 | 361955.88 |
| Alanine-13C3 15N |  |  |  |  | 6003538.94 | 5324598.01 |
| L-Aspartic Acid |  |  |  |  | 220458.70 | 206334.14 |
| L-Proline |  |  |  |  | 74461.08 | 28259.86 |

**Supplementary Table 3**. Amino Acids quantified in Wet Plasma and Dried Plasma Spots (Parafilm and Noviplex^TM^). STDEV (Standard Deviation); WP (Wet Plasma); PF (Parafilm); NP (Noviplex^TM^)
